# Heart failure burden and care among cardiology inpatients: insights from the Hellenic Cardiorenal Morbidity Snapshot (HECMOS) study

**DOI:** 10.1007/s00392-025-02610-x

**Published:** 2025-04-22

**Authors:** Ioannis Leontsinis, Sotiria Liori, Dimitrios Farmakis, Aggeliki Valatsou, Panagiotis Vlachakis, Christina Antonia Verikokou, Ioanna Delia Vlad, Vasileios Giannaris, Georgios Giannopoulos, Kyriakos Dimitriadis, Panagiotis Theofilis, Georgios Karakostas, Christoforos Komporozos, Georgios Konstantinides, Stylianos Lambropoulos, Aikaterini Lionti, Athanasios Makris, Panteleimon Makridis, Ioannis Mamarelis, Maria Marketou, Aikaterini Naka, Georgios Nikitas, Periklis Davlouros, Evaggelos Oikonomou, Nikolaos Papaioannou, Athanasios Pipilis, Assaf Sawafta, Pavlos Skantzikas, Michael Siarkos, Theodoros Sinanis, Konstantinos Skordis, Georgios Spiromitros, Ioannis Stamoulopoulos, Konstantinos Tsatiris, Dimitrios Tsiachris, Michael Fosteris, Emmanouel Foukarakis, Chistina Chrysochoou, Gerasimos Filippatos, Konstantinos Tsioufis

**Affiliations:** 1 First Cardiology Department, School of Medicine, Hippokration General Hospital, National and Kapodistrian University of Athens, 114 Vass Sofias Ave, 11528 Athens, Greece; 2 School of Medicine, Department of Cardiology, National and Kapodistrian University of Athens, Attikon University Hospital, Athens, Greece; 3 General Hospital of Athens “G.Gennimatas” Hospital, Athens, Greece; 4 Cardiology Department, Aghios Panteleimon General Hospital, Nikaia, Greece; 5 Thriasio General Hospital, Elefsina, Greece; 6 Hippocratio General Hospital, Thessaloniki, Greece; 7 Kilkis General Hospital, Kilkis, Greece; 8 Athens Naval Hospital, Athens, Greece; 9 Cardiology Department, General Hospital of Drama, Drama, Greece; 10 Mamatsio General Hospital, Kozani, Greece; 11 KAT General Hospital, Athens, Greece; 12 Cardiology Department, General Hospital of Argos, Argos, Greece; 13 Cardiology Department, General Hospital of Edesa, Proastio, Greece; 14 401 Athens Military Hospital, Athens, Greece; 15 Department of Cardiology, Heraklion University Hospital, Iraklio, Crete Greece; 16 University Cardiology Clinic, University of Ioannina, Ioannina, Greece; 17 Panarkadiko General Hospital, Tripoli, Greece; 18 Cardiology Department, Patras University Hospital, Rion, Greece; 19 3rd Cardiology Department, University of Athens, Sotiria Hospital, Athens, Greece; 20 Department of Cardiology, Asclepieion Hospital, Athens, Greece; 21 Hygeia General Hospital, Athens, Greece; 22 Department of Cardiology, University General Hospital of Larissa, Larissa, Greece; 23 Cardiology Department, “Tzaneio” General Hospital of Piraeus, Piraeus, Greece; 24 Cardiology Department, G. Papanicolaou Hospital, Thessaloniki, Greece; 25 Cardiology Department, Chania General Hospital, Chania, Greece; 26 Arta General Hospital, Arta, Greece; 27 Katerini General Hospital, Katerini, Greece; 28 Karditsa General Hospital, Karditsa, Greece; 29 Sparti General Hospital, Sparti, Greece; 30 Venizelio-Pananio General Hospital, Heraklion, Greece

**Keywords:** Chronic heart failure, Heart failure unit, Acute heart failure, Guideline-directed medical treatment, Comorbidities

## Abstract

**Purpose:**

Heart failure (HF) burden and care varies significantly across different countries. We aimed to illustrate the clinical characteristics and HF-related care among cardiology inpatients in Greece.

**Methods:**

We collected information about all cardiology inpatients on the 3rd of March 2022. The current analysis focuses on acute or chronic HF.

**Results:**

Among a total of 923 participants, 280 (30%) concerned cases of acute HF whereas 351 patients (38%), (median age 79 ± 12 years, male gender 63.8%) had a history of chronic HF, with their majority presenting with multiple comorbidities and previous HF hospitalizations. 173 (49%) of chronic HF participants had reduced LVEF. Ischemic heart disease was the predominant HF etiology (182, 51.9%). Prior to the index admission, chronic HF cases were receiving diuretics, beta blockers, ACEi/ARBs, ARNI, MRAs, and SGLT2i at 79.8%, 74.4%, 43.3%, 10.8%, 40.7%, and 14%, respectively. Independent predictors of lower prescription rates of Guideline Directed Medical Therapy (GDMT) included advanced age (*p* < 0.001), chronic kidney disease (RASi OR 0.392, *p* = 0.008, MRA OR 0.523 *p* = 0.097), and lack of follow-up in dedicated HF clinics (*p* = 0.006). No regional differences with regards to GDMT were identified.

**Conclusion:**

In this nation-wide real-world snapshot study, patients with chronic and acute HF accounted for a significant proportion of cardiology inpatients, while ischemic heart disease was the leading HF cause. GDMT and device therapy can be improved. Follow-up in dedicated HF units was related with increased prescription rates of GDMT, whereas this was not affected by geographical region.

**Graphical Abstract:**

Chronic heart failure regional distribution (regional density of participants visualized on a map with person-shaped markers), comorbidities, and pharmacotherapy. HECMOS

**Figure:**
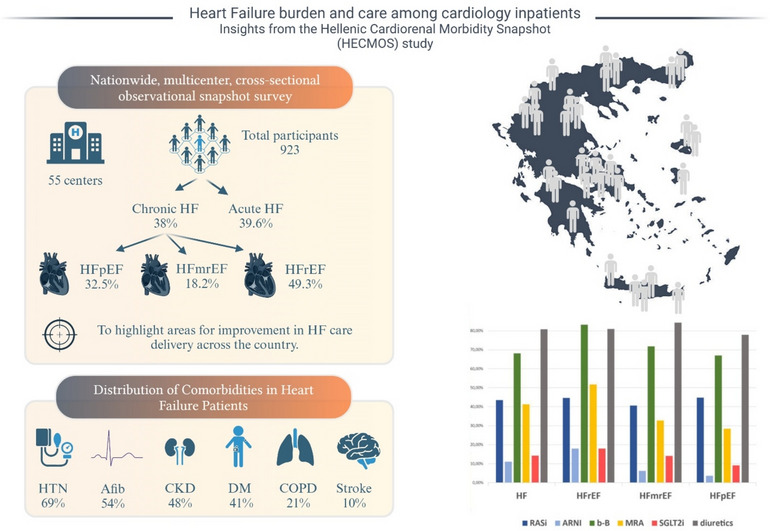

## Introduction

Heart failure (HF) burden, patient characteristics, and treatment strategies vary significantly among regions, reflecting several factors such as lifestyle habits, socioeconomic status, genetic predisposition, and accessibility to health services [1, 2]. According to recent epidemiological data, HF incidence and prevalence in Greece is estimated at 4 per 1000 patient-years and 10 per 1000 persons, respectively [3]. The decline in birth rates in conjunction with the increase in life expectancy has resulted in a rapidly aging Greek population. Cardiovascular morbidity, being affected by the recent economic crisis, the COVID-19 pandemic, and the rather high burden of cardiovascular risk factors, despite a previous recession, have shown similar increasing trends [4, 5]. Furthermore, the constantly increasing recognition of HF with preserved ejection fraction (HFpEF) as well as the striking disturbance of HF-related services after the COVID-19 pandemic may have influenced HF care in Greece [6, 7].

The Hellenic Cardiorenal Morbidity Snapshot (HECMOS) study was conducted after the fourth wave of the COVID-19 pandemic and was designed to depict the contemporary trends of cardiorenal morbidity among cardiology inpatients in Greece. The current paper specifically focuses on HF, offering a cross-sectional nation-wide report on HF burden, its clinical characteristics, and relevant comorbidities. It also aims at delineating HF therapy trends, to determine potential inertia in guideline-directed medical or device treatment implementation and to explore the potentials and needs for novel therapies, and thus in additional healthcare expenditure.

## Methods

HECMOS is a multicenter cross-sectional observational snapshot survey which investigated the contemporary trends of cardiorenal morbidity among hospitalized patients in cardiology wards across Greece on an ordinary weekday, the 3rd of March 2022. The study included 923 cases from 55 different departments in total, adequately covering the whole country given its geographic peculiarities. Eligibility for enrollment was offered to all adult inpatients able to provide informed consent by themselves or via their legal representative. The HECMOS study was organized by the 1 st Department of Cardiology of the National and Kapodistrian University of Athens (NKUA) in collaboration with the 2nd and 3rd NKUA Cardiology department under the auspices of INAKEN (Institute for study, research, education of vascular, heart, brain, and kidney nosologies).

Data collection happened in a de-anonymized manner and included patients’ demographics, physical characteristics and lifestyle parameters. Details regarding the participants’ past medical history were gained with emphasis on the presence of chronic HF, atrial fibrillation (AF), chronic kidney disease (CKD), diabetes mellitus (DM), hypertension, Covid-19 infection, stroke, severe liver dysfunction, bleeding history, chronic obstructive pulmonary disease (COPD), obstructive sleep apnea syndrome (OSAS), and intracardiac devices such as a pacemaker (PPM) or a defibrillator (IDC). Chronic HF cases were further interrogated about the left ventricular ejection fraction (LVEF) range, the right ventricular systolic capacity, the underlying substrate of cardiac dysfunction and any prior risk assessment for sudden cardiac death. Regular follow-up in dedicated heart failure units was also noted if present. For all non-elective admissions, a detailed description was obtained which included symptoms and sings on admission as well as the working diagnosis on the day of the snapshot survey. Data collection included electrocardiographic, echocardiographic and laboratory results synchronous to the snapshot survey. Laboratory results included hemoglobulin, INR, uric acid, total cholesterol, HDL, LDL, triglycerides, iron studies, TSH, liver function studies, glycosylated hemoglobulin (HbA1 C), fasting glucose, maximum troponin, natriuretic peptides, and high sensitivity CRP levels, whereas serial values of urea, creatinine, sodium and potassium levels were also available. eGFR was calculated according to the 2021 CKD-EPI equation. A detailed medication and vaccination history was collected together with information about the participants’ adherence and any previous hospitalization events. In addition, a comprehensive list of invasive or intravenous treatments administered during the index admission until the date of the survey was obtained together with all medications offered on the 3rd of March 2022.

### Definitions

In this study, HF definition and its further classification into the subtypes of HFrEF, HFmrEF, HFpEF, and isolated right ventricular failure were in accordance to the latest ESC guideline (2021) [8]. Patients with acute HF with no previous history of HF were classified as new HF diagnosis cases. HF cases were further divided into groups with regards to the region of being hospitalized in. Centers located in Athens, Thessaloniki, Patras, Larisa, Heraklion, Ioannina, and Alexandroupoli were classified as urban, whereas the rest of the participating canters as rural. In the same lines, AF and acute coronary syndrome (ACS) cases were classified according to the 2020 respective ESC guidelines [9, 10]. Hypertension was defined in accordance to the 2018 ESH/ESC guidance [11]. The classification of DM and pre-DM was based on the WHO 2019 recommendations [12]. Finally, CKD definition in the current study required the presence of a stage 3 chronic kidney impairment (eGFR < 60 ml/min/1.73 m2) on at least two different measurements into a minimum period of 3 months prior to enrollment [13].

All data (supplementary section) were recorded anonymously in a pre-specified eCRF. This form has been created through the online RedCap platform (Vanderbit University). The study protocol was in accordance to the 1975 declaration of Helsinki, and it was approved by the local Ethical Committees of each of the participating institutions (10/11/2021-No 4358). The anonymity and confidentiality of the collected data were ensured in accordance with the applicable legislation.

### Statistics

Continuous variables are presented as mean ± standard deviation or median with the respective interquartile range. Categorical variables are described as counts with percentages. Comparison of continuous variables was performed using two sample *t* test and Mann–Whitney test, as appropriate. Differences in categorical variables were tested with the use of X2 test. Univariate and multivariate logistic regression analyses were performed to identify predictors of adherence to guideline-directed medical treatment (GDMT). All statistical analyses were performed using SPSS 23.0 software package (SPSS Inc., Chicago, IL, USA). A two-sided *P* value less than 0.05 was considered statistically significant.

## Results

### Chronic heart failure

The HECMOS study enrolled a total of 923 patients admitted on the 3rd of March, 2022 at 55 hospitals across the country [14]. Among them, 351 (38%) with a history of chronic HF (median age 79 ± 12 years, male gender 63.8%) were included (Graphical abstract). Most of HF patients (298, 84.9%) were admitted on an urgent basis after visiting the emergency department. Among HF patients, 173 cases (49.3%) had HFrEF, 64 (18.2%) had HFmrEF, and 114 (32.5%) HFpEF. In addition, 78 (22.2%) patients had a diagnosis of concomitant significant right ventricular dysfunction, whereas 4 (1.1%) had isolated right ventricular failure. 56.1% of the sample reported episodes of HF hospitalization during the previous 12-month period, while 20.8% had regular follow-up in a dedicated HF unit (11.9%, 20.3%, and 27.1% of HFpEF, HFmrEF, and HFrEF, respectively). Ischemic heart disease (IHD) was the predominant underlying HF etiology, followed by valvular and hypertensive heart disease, accounting for 53.1%, 24.2%, and 14.8% of patients, respectively.

In terms of medical history, hypertension was highly prevalent accounting for 68.7% of cases; 190 patients (54.1%) had atrial fibrillation (AF) [134 (70.5%) permanent/persistent AF, 56 (29.5%) paroxysmal AF (PAF)], among whom 3.1% had previously received AF ablation. Chronic kidney disease (CKD) and diabetes mellitus (DM) accounted, respectively, for 47.9% and 41.3% of HF patients (Table 1). Among HF patients, 43.9% had fulfilled the recommended vaccination scheme including streptococcus pneumoniae and seasonal flu, and 79.5% reported complete vaccination against the COVID-19 disease (Appendix Fig. 1).

**Table 1:** Chronic HF: HECMOS

	*N* = 351 (%)
Unscheduled admission	298 (84.9%)
Previously admitted for HF	197 (56.1%)
Follow-up in HF units	73 (20.8%)
Demographics—physical characteristics	
Gender, male	224 (63.8%)
Age	Mean (SD) 79 (± 12) years
BMI	Mean (SD) 26.8 (± 5.3) kg/m^2^
Lifestyle factors	
Current smoking	51 (14.5%)
Any physical activity, min/w	63 (17.9%)
Alcohol consumption (daily/occasionally)	120 (34.2%)
Past medical history	
Hypertension	241 (68.7%)
AF (chronic and paroxysmal)/chronic AF	190 (54.1%)/134 (38.2%)
Previous AF ablation	*6 (3.1*%*)*
CKD	168 (47.9)
Previous MI, peripheral artery disease, revascularization	173 (49.3%)
DM	145 (41.3%)
COPD	72 (20.5%)
Ischemic stroke/TIA	36 (10.2%)
Bleeding history with high risk for recurrence	27 (7.8%)
OSAS/CPAP users	19 (5.4%)/7
Significant liver dysfunction	10 (2.8%)
Adherence to medication	193 (55%)
Vaccination history	
Covid-19 (complete including booster dose)	279 (79.5%)
Influenza and Str pneumoniae	154 (43.9%)
Influenza without Str pneumoniae	70 (19.9%)
Str pneumoniae without influenza	10 (2.8%)
Neither influenza nor Str pneumoniae	104 (29.6%)
Basic laboratory results	
Pulmonary arterial systolic pressure (PASP) (mmHg)	46 ± 14
Urea (mg/dl)	80 ± 43
Creatinine (mg/dl)	1.1 (IQ1 0.9–IQ3 1.6)
eGFR (ml/min/1.73^2^)	34 (IQ1 14–IQ3 65)
Potassium (mmol/L)	4.3 (IQ1 4–IQ3 4.7)
Sodium (mmol/L)	139 (IQ1 136–IQ3 141)
HbA1c (%)	6.1 (IQ1 5.6–IQ3 6.8)
Hb (g/dL)	12.9 (IQ1 11–IQ3 14.4)
BNP pg/ml	390 (IQ1 107–IQ3 1281)
proBNP pg/ml	2456 (IQ1 764–IQ3 9725)
Ferritin μg/L	106 (IQ1 42–IQ3 224)
CRP mg/L	7.8 (IQ1 2–IQ3 26)
Medications before index admission	All (*n* = 351)
Diuretics	280 (79.8%)
B-blockers	261 (74.4%)
OAC	179 (51%)
RASi	152 (43.3%)
ARB	(49.0%)
ACEi	(51.0%)
Anti-platelets	148 (42.2%)
MRAs	143 (40.7%)
Anti-diabetic drugs	119 (33.9%)
Uric acid lowering drugs	102 (29.1%)
CCBs—DHP	79 (22.5%)
Other antiarrhythmics	59 (16.8%)
SGLT2i	49 (14%)
ARNI	38 (10.8%)
CCBs—non DHP	9 (2.6%)
Devices	
ICD	36 (10.3%)
PPM	33

Main demographic and clinical features

Concerning pharmacotherapy before the index admission, diuretics were the most common medications irrespective of LVEF (78.6%, 84.4%, 78.9%, respectively, *p* = 0.61). The most commonly used diuretic agent was furosemide, while six patients (1.7%) were receiving diuretic combinations (loop diuretic together with thiazide or thiazide-like). Beta blockers (b-blockers) were being received by 74.4% of cases, and their prescription rate was significantly higher in HFrEF, reaching 80.9% (*p* = 0.026). Angiotensin-converting enzyme inhibitors (ACEi) or angiotensin receptor blockers (ARBs) had been prescribed to 43.3% of the chronic HF cases. There was no statistically significant difference in ACEi/ARB usage with regards to LVEF; however, HFrEF cases were receiving more often ACEi rather than ARBs (64% vs 36%), while HFpEF patients were more likely to receive ARBs (65.3% vs 34.7%) (Table 2). Angiotensin receptor/neprilysin inhibitors (ARNI) were being received by 17.3%, 6.2%, and 3.5% of the HFrEF, HFmrEF, and HFpEF subgroups, respectively. Mineralocorticoid receptor antagonists (MRA) were being received by 40.7% of patients; HFrEF cases were more likely to receive MRAs than HFpEF ones (50.3% vs 30.7%, *p* = 0.001), whereas most of the patients were receiving eplerenone (68.3%). Among HFrEF patients who were not on MRA on admission (n = 86, 49.7%), their median eGFR and potassium levels were 41.75 [interquartile range (IQR), 25.48–62.07 ml/min/1.73 m^2^] and 4.4 mmol/L (IQR, 3.9–4.8 mmol/L), respectively. Sodium–glucose co-transporter 2 inhibitors (SGLT2i) had been prescribed in 71 patients (14%), including 17.3% of HFrEF and 8.8% of HFpEF (p = 0.16); 85.9% of patients on SGLT2i were diabetic and 57% had CKD. Among HFrEF patients, 42.7% had been previously evaluated for sudden cardiac death (SCD) risk stratification, whereas implantable cardioverter defibrillator (ICD) and cardiac resynchronization therapy (CRT) had been offered to 19.7% and 0.03%, respectively. In addition, 35.4% of the HFrEF cases would potentially be candidates for CRT assuming that LVEF was ≤ 35% and based on the recent European Society of Cardiology guidance [15] (Appendix Fig. 2).

**Table 2:** Chronic HF: HECMOS

	HFrEF (*n* = 173)	HFmrEF (*n* = 64)	HFpEF (*n* = 114)
Diuretics	136 (78.6%)	54 (84.4%)	90 (78.9%)
B-blockers	140 (80.9%)	44 (68.8%)	77 (67.4%)
OAC	73 (42.2%)	29 (45.3%)	77 (67.4%)
RASi	76 (43.9%)	26 (40.6%)	50 (43.9%)
*ARB*	(36.0%)	(57.7%)	(65.3%)
*ACEi*	(64.0%)	(42.3%)	(34.7%)
Anti-platelets	88 (50.9%)	28 (43.8%)	23 (20.2%)
MRAs	87 (50.3%)	21 (32.8%)	35 (30.7%)
Anti-diabetic drugs	61 (35.3%)	20 (31.2%)	38 (33.3%)
Uric acid lowering drugs	46 (26.6%)	21 (32.8%)	35 (30.7%)
CCBs—DHP	24 (13.9%)	18 (28.1%)	37 (32.5%)
Other antiarrhythmics	34 (19.7%)	13 (20.3%)	12 (10.5%)
SGLT2i	30 (17.3%)	9 (14%)	10 (8.8%)
ARNI	30 (17.3%)	4 (6.2%)	4 (3.5%)
CCBs—non DHP	4 (2.3%)	2 (3.1%)	3 (2.6%)
ICD	34 (19.7%)	2 (3.1%)	0
PPM	15 (8.7%)	10 (15.6%)	8 (7%)

Treatment offered before index admission per EF category

### Predictors of GDMT utilization among HFrEF and HFmrEF cases

Advanced age was found to be inversely related with the concomitant utilization of all four disease-modifying medications in HFrEF and HFmrEF cases (*p* < 0.001). In the same lines, patients with larger left ventricular end diastolic diameter and those been followed up in dedicated HF units were found to receive GDMT in higher proportions (*p* < 0.005 and *p* = 0.006, respectively). The presence of comorbidities (DM, AF, CKD), BMI levels, or male gender were not associated with the concomitant utilization of all four categories of GDMT. However, patient receiving three out of four foundational therapies were less likely to have CKD (OR 0.38, IQR 0.159–0.93, *p* = 0.0034) but more often diagnosed with dilated cardiomyopathy (OR 5.4, IQR 1.66–17.64, *p* = 0.005). With regards to each of the GDMT categories, age was inversely related with utilization rates of ARNI (OR 0.961: *p* = 0.073), MRAs (OR 0.956: *p* = 0.006), and SGLT2i (OR 0.941: *p* = 0.016). Male HF patients were less likely to receive MRAs than the female ones (OR 0.512: *p* = 0.098). Moreover, history of AF was found to be related with increased utilization rates of b-blockers and MRAs (*p* = 0.056 and *p* = 001, respectively). In addition, patients with CKD received ACEi/ARB/ARNI or MRAs less frequently (*p* = 0.008 and *p* = 0.097, respectively), whereas HFrEF/HFmrEF cases with DM were more likely to receive MRAs or SGLT2i (*p* = 0.049 and *p* = 0.001, respectively). No significant trend with regards to HFrEF/HFmrEF etiology and GDMT utilization was found, apart from DCM cases receiving MRAs and SGLT2i in higher proportion than the ones with IHD (*p* = 0.004 and *p* = 0.05, respectively) and valvular HF cases receiving ACEi/ARB/ARNI less often than the ones with ischemic origin (*p* = 0.048) (Appendix Tables 2, 3). Participants were asked to provide information about adherence to medication. A multivariate logistic regression analysis revealed that a history of DCM tended to independently predict higher adherence to medication (OR 5.292, IQR 0.857–32.678, *p* = 0.073). We found no significant correlation between reported adherence levels and age, gender or a history of DM, CKD, AF, prior HF hospitalization events or follow-up in HF units (Appendix Table 3).

### Comparison between urban and rural area hospitals

We did not find any statistically significant difference with regards to GDMT utilization between HFrEF cases admitted in urban versus rural areas. Moreover, chronic HF patients in urban and rural areas had similar trends of previous hospitalizations for HF exacerbation across the LVEF spectrum and similar possibility of being followed up in dedicated HF units (Table 3).

**Table 3:** HECMOS; regional differences in chronic HF cases

	Urban areas (ν = 269) *Ν* (%)	Rural areas (ν = 82) *Ν* (%)	*p* value
Follow-up in dedicated HF unit	58(22.0%)	15 (18.3%)	0.476
Previous HF hospitalization	147 (55.7%)	50 (62.5%)	0.280
HFrEF: SCD risk stratification	64 (45.1%)	9 (31.0%)	0.164
Prior to admission treated with			
RASi (HFrEF)	66 (45.8%)	10 (34.5%)	0.261
ARNI (HFrEF)	27 (18.8%)	3 (10.3%)	0.275
MRA (HFrEF)	77 (53.5%)	10 (34.5%)	0.062
SGLT2i	42 (15.6%)	7 (8.5%)	0.106

### Impact of follow-up in dedicated HF units

ARNI (31.9% vs 12.1%, *p* = 0.002) and MRA (66% vs 43.6%, *p* = 0.009) but not ACEi/ARB (36.2% vs 46.8%, *p* = 0.2) utilization rates were higher among HFrEF cases having been followed up in dedicated HF units. Along those lines, HFrEF cases with follow-up in HF units were more likely to have undergone SCD risk stratification (80.9% vs 27.1%, *p* = 0.001). Furthermore, chronic HF cases with previous follow-up in HF units demonstrated higher rates of previous HF hospitalizations (84.5% vs 50%, *p* = 0.01) and higher rates of SGLT2i utilization (31.5% vs 9.5%, *p* = 0.01) irrespective of LVEF phenotype (Table 4).

**Table 4:** HECMOS; heart failure units contribution in chronic HF management

Follow-up	No dedicated HF unit (*n* = 273)	Dedicated HF unit (*n* = 73)	*P* value
Previous HF hospitalization	134 (50.0%)	60 (84.5%)	**0.001**
HFrEF: SCD risk stratification	33 (27.1%)	38 (80.9%)	**0.001**
Prior to admission treated with			
RASi (HFrEF)	58 (46.8%)	17 (36.2%)	0.212
ARNI (HFrEF)	15 (12.1%)	15 (31.9%)	**0.002**
MRA (HFrEF)	54 (43.6%)	31 (66.0%)	**0.009**
SGLT2i	26 (9.5%)	23 (31.5%)	**0.001**

*P*-values < 0.05 are given in bold

### Acute heart failure

The HECMOS study enrolled 280 cases being admitted with acute HF, representing 39.6% of the total non-elective admissions. Among them, 72 cases (25.7%, male gender 70.8%, age 70 ± 14.7 years) concern patients with a new diagnosis of HF (de novo HF), whereas 208 patients (74.3%, male gender 63%, age 65 ± 14.9 years) were hospitalized for HF exacerbation (decompensated HF). Most of the cases in both subgroups presented with reduced LVEF (51.4% and 47.6%, respectively). Among de novo HF cases, the most frequent underlying trigger was hypertensive emergency (40.6%), followed by acute coronary syndrome (21.7%) and tachyarrhythmias (18.8%). On the other hand, among cases with decompensated HF, the most commonly recognized triggers included non-adherence to dietary restriction (33%) or chronic HF medication (27.1%) and acute kidney injury—cardiorenal syndrome (26.9%). Hypertension and DM were more prevalent among de novo as compared to decompensated HF. On the other hand, patients with decompensated HF suffered more frequently from AF, CKD, and COPD.

During the index admission for acute HF, patients with de novo HF and decompensated HF were similarly treated with diuretics (88.9% vs 95.7%), ARNI (9.7% vs 10.6%), b-blocker (76.4% vs 75%), and SGLT2i (19.4% vs 21.6%). However, ACEi/ARB treatment was more frequently offered to cases with de novo HF (ACEi: 27.8% vs 19.7%, ARBs: 29% vs 14.9%). Inotrope utilization was more frequent among patients with decompensated HF (22.1% vs 15.3%) as was renal replacement therapy (16.3% vs 5.6%). (Appendix, Table 1).

### Eligibility of additional treatment in cases admitted with decompensated HF

79% of cases admitted with decompensated HF despite being eligible for SGLT2i treatment prior to admission were not receiving such therapy. On snapshot day, the proportion of cases not on SGLT2i dropped to 68% signifying that this index admission triggered this treatment initiation. Similarly for decompensated HFmrEF/HFrEF, the rates of non-utilization of MRAs dropped from 56 to 44%, whereas for b-blockers, ARNI and ACEi/ARB increased (20% to 24%, 85% to 86% and 63% to 85%, respectively). Iron deficiency was identified among 29% of the acutely decompensated cases, but intravenous iron supplementation was offered only to 5% of those.

Finally, 76.6% of the decompensated HFrEF cases could benefit from the addition of vericiguat to their post-discharge treatment.

## Discussion

The HECMOS study was the first nation-wide survey to document the cardiorenal morbidity among cardiology inpatients in Greece, and the current analysis focuses on real-world data regarding HF. A detailed description of all comorbid conditions and current therapeutic strategies applied, among the sicker subgroup of the HF population that is those being admitted to a cardiology department, are described in the present analysis.

Chronic HF was highly prevalent concerning 38% of all cardiology inpatients. Male gender, reduced LVEF, limited physical activity, previous HF hospitalizations, older age, and IHD concerned the majority of the cases. HFpEF patients were older than the ones with reduced LVEF, whereas this subgroup included more women than men. The aforementioned findings come in agreement with similar studies [16], and relevant population-based epidemiological data [5, 17, 18]. Chronic HF phenotyping across our sample is further detailed by several comorbid conditions. Interestingly, HFpEF cases had higher rates of preexisting hypertension, AF, COPD, and OSAS. On the contrary, a history of stroke, DM, and CKD was more prevalent in patients with HFrEF. Uncontrolled hypertension concerned 24% of the hypertensive cases, being more prevalent among the HFpEF population. Adequate blood pressure control contributes in HF prevention but also improves prognosis, particularly in HFpEF [19]. Hypertensive emergencies were the commonest triggering factor for de novo HF hospitalizations, a finding suggestive that adequate blood pressure control in this particularly sick population remains a rather unmet need. AF was the second most prevalent comorbid condition. Interestingly, the proportion of patients ever treated with AF ablation was 3.1%. HF and AF interconnection has been thoroughly studied [20] and AF ablation improves prognosis in HF [20, 21]. Although these procedures gain grounds over time [22], our results suggest that they remain rather underused and COVID-related disruption may have contributed in this. Third in relevance, CKD concerned most of the patients and was found to negatively affect the GDMT utilization rates.

On the other hand, acute HF cases were also common, accounting for 40% of all non-elective admissions with their majority to concern events of HF decompensation. Non-adherence to dietary restrictions and/or medication was found to contribute to the majority of these events. It is not uncommon for HF patients to fail to persist in the prescribed treatment, and this in not only driven by side effects but also related to comorbid conditions like CKD [23]. Contrarily to previous reports [24], we found no significant correlation between adherence to medication and advanced age. Arguing that HF decompensation events are usually multifactorial, we speculate that promoting adherence would have a positive impact on hospitalization events.

Chronic HF cases offered information about regular HF care such as GDMT and immunization. The low vaccination rate against common respiratory infections can be partially justified by the unprecedented need to confront the high mortality burden of COVID-19; however, it highlights a shift of interest away from the standard care, arguably harmless in a highly morbid population.

Regarding GDMT and its implementation rates in different phenotypes, most of the chronic HF patients were receiving diuretics on a regular basis which indirectly implies a rather compromised functional capacity. Overall b-blocker, ACEi/ARB/ARNI, MRA, and SGLT2i prescription rates were 74.4%, 54.1%, 40.7%, and 14%, respectively, and these were inversely related to the LVEF range. It is important to highlight that when the study was conducted, SGLT2i recommendation concerned HFrEF as well as diabetic patients with HF.

Previous national data from the ALARM-HF (2006–7) and the ESC-HF pilot survey (2009–2010) [25] reported increase in ACEi/ARB, b-blocker and MRA utilization rates from 47%, 31%, and 18% to 60%, 65%, and 45%, respectively. We demonstrate even higher b-blocker rates with MRA and ACEi/ARB/ARNI utilization to decline. Despite differences in design, the signal of improvement in b-blocker utilization is definitely encouraging. However, underutilization of ACEi/ARB/ARNI and MRA warrants consideration. Focusing on chronic HFrEF, that GDMT grants the stronger recommendation, our results compared with the ESC-HF long term ones [26] illustrate the same trend. Compromised renal function or hyperkalemia could contribute to these findings. Indeed, half of the participants suffered from CKD. Novel potassium binders have been shown to increase ACEi/ARB tolerance [27]; however, the availability of such products in Greece were limited. Nevertheless, among the HFrEF cases not on MRA on admission, we identified several cases with no absolute contraindication, concluding that MRA utilization was rather suboptimal. However, CKD and related hyperkalemia were not the only reasons for abstinence from MRAs. Male gender was significantly related with lower odds of this treatment. Finerenone, proven to be efficacious in HFrEF [28] but also to allow treatment in CKD patients with lower risk of hyperkalemia and at the same time to express beneficial effects on kidney function [29], carries minimum anti-androgenic properties and is expected to enter the HF field during the next years. In our sample, half of the HFrEF cases were not receiving MRA. Given the high prevalence of CKD, we assume that finerenone treatment would be a therapeutic proposal in such selected cases. Nevertheless, focusing on currently available medication quiver, MRA treatment presents high discontinuation trends [30]; therefore, effort should be taken not only to initiate but also to maintain treatment in higher acceptable dosage.

Age was another important contributor to GDMT. Focusing on reduced LVEF cases (< 50%) in whom the synchronous to the study recommendations clearly supported the so called four pillars of treatment (i.e., ACEi/ARB/ARNI, MRAs, b-blockers and SGLT2i), we demonstrated that as the age advanced, the sum of drug categories each patient was receiving declined. The negative effect of advanced age on GDMT prescription rates was mainly driven by lower odds of ARNI, MRA, and SGLT2i. This comes in agreement with previous reports [31] and can be attributed to multiple factors such as frailty and multi-morbidity which usually accompany advanced age. Although the elderly population has generally been excluded from the large RCTs, evidence to support an age-related lower benefit from GDMT in HF is lacking. On the other hand, as adherence reporting was independent to age, it seems reasonable to think that GDMT underutilization among the elderly participants was not likely related to non-adherence. Therefore, the efforts to offer disease-modifying therapy to all eligible HF population should be reinforced.

Another important aspect regarding GDMT implementation in chronic HF concerns the low utilization rates of SGLT2i (14% of all chronic HF cases). Prescription rates were higher among the HFrEF subgroup compared to HfmrEF/HfpEF. Interestingly, the vast majority of cases on SGLT2i were diabetic, a finding implying that their primary indication was DM rather than HF. This can be explained as reimbursement for SGLT2i in non-diabetic patients was granted 1 month prior to the study and at that time and this was limited to HfrEF. On the contrary, ARNI, RASi, b-blocker, and MRA therapy reimbursement was established years before the conduction of the study. Meanwhile, the class I recommendation for SGLT2i [32] treatment irrespectively of LVEF and diabetes was offered after the accomplishment of our study. However, the beneficial effects on diabetic subjects were well established, and as DM concerned 41.3% of our sample, we expected SGLT2i penetration to be higher. Nevertheless, in-hospital SGLT2i utilization increased, implying that physicians’ alertness is improving.

To the best of our knowledge, this is the first nation-wide report on chronic HfpEF treatment among cardiology inpatients. As compared with the GDMT utilization rates of EMPEROR-Preserved and DELIVER clinical trials, the HECMOS HfpEF patients received all three categories at lower rates. Besides, our sample is much smaller and includes older patients with worse indices of renal function. Similar findings appear after comparing HFrEF cases with the relevant trials. Optimal care in HFrEF is not limited to GDMT. ICD implantations concerned 19.7%, whereas we identified several cases who would benefit from CRT. Our results suggest that device therapy and GDMT implementation in Greece, particularly with regards to HFrEF, can be improved on a national level.

Beyond renal dysfunction, there are several reasons which jeopardize GDMT in HF, namely financial, geographical, and physician inertia [1, 30, 33]. GDMT was similar between urban and rural areas. On the contrary, regular follow-up in HF units was related with higher ARNI and MRA prescription rates among HFrEF cases, and higher SGLT2i rates across the LVEF spectrum. Reasonably, dedicated HF teams can improve quality of care in HF patients with direct implications on quality of life and cost [34]. The HF care in Greece is being transformed [7]. Given its geographic peculiarities, our results dictate the necessity of adopting modern technologies and switching the physician-centered current map to the more successful multi-disciplinary approach.

Our study is the first to provide nation-wide data on clinical characteristics, comorbidities, and treatment strategies for cardiology inpatients with chronic and acute HF across the whole LVEF range in Greece. Nevertheless, these results should be viewed under the study’s limitations. Snapshot cross-sectional design offered an abundance of data in a short period of time; however, it did not include information about the duration of hospital stay, short- and long-term follow-up. Furthermore, despite the broad geographical distribution, the HECMOS study addressed only cardiology inpatients, thus not having included HF, which is often the case in HFpEF. Seasonal variations of HF admission throughout the year are not reflected either. Within the dataset, LVEF was documented in ranges set by the current ESC-HF guidelines. Consequently, cases with LVEF < 35% were not identifiable among the HfrEF subgroup (LVEF < 40%). For this reason, CRT candidates are declared as potential ones, with the exception of 18 cases with definite CRT indication (permanent RV pacing).

## Conclusion

In this nation-wide, real-world, snapshot study, patients with HF accounted for a significant proportion of cardiology inpatients. IHD was the leading HF cause. Although b-blocker implementation rate appears satisfactory according to previous data, other pharmacological therapies or device therapy appeared to be suboptimally offered. GDMT was not influenced by geographical region but age and cardiorenal morbidity were significantly related with lower implementation rates, whereas follow-up in dedicated HF units showed the amelioration of the latter. Our study underscores the need to increase guidelines proposed treatment strategies implementation through better education and training. Snapshots like HECMOS provide the “tools’ to comprehend current limitations in HF therapy in the healthcare system and identify the means to improve cardiovascular care in high-risk and cost patient settings.

## Figures and Tables

**Fig. 1:**
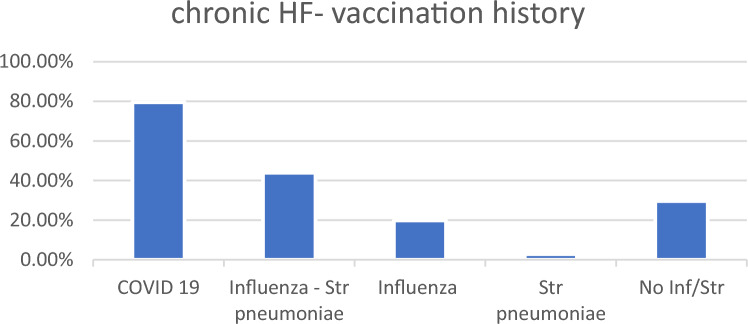
HECMOS: Chronic HF—vaccination history

**Fig. 2:**
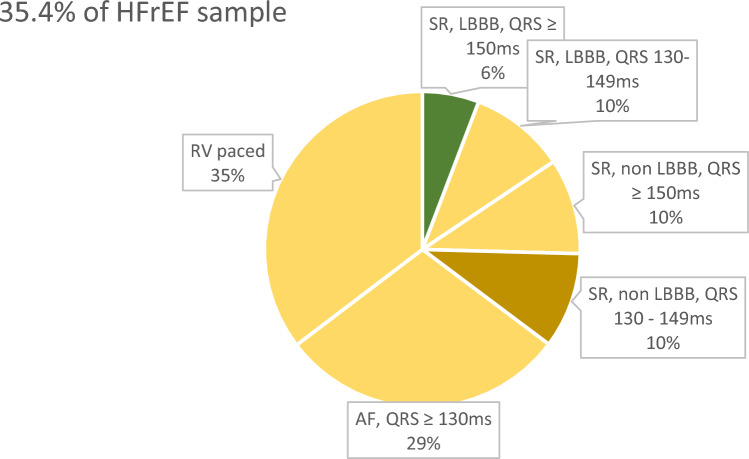
HFrEF with no CRT and permanent Right ventricular pacing or conduction delay abnormality

## Data Availability

Data is available from the corresponding author upon reasonable request.

## Appendix (see Figs. 1, 2 and Tables 5, 6, 7)

**Table 5:** Acute HF: clinical characteristics and inpatient management

Acute HF—HECMOS	New HF diagnosis N = 72		HF exacerbation N = 208	
	Ν (%)		Ν (%)	
Demographics—physical characteristics				
Gender, male	51 (70.8%)		131 (63%)	
Age	70 ± 14.7		65 ± 14.9	
BMI	28 ± 4.9		27 ± 4.9	
Lifestyle factors				
Currently smoking	23 (31.9%)		25 (12%)	
Any physical activity, min/w	22 (30.5%), 120 (Q1 60, Q3 210)		25 (12%), 120 (Q1 60, Q3 120)	
Alcohol consumption (daily/occasionally)	13 (18%)/24 (33%)		7 (3.3%)/59 (28.4%)	
Past medical history				
Hypertension	50 (69.4%)		93 (44.7%)	
AF (chronic and paroxysmal)/chronic AF	12 (16.7%)/7 (9.7%)		134 (64.4%)/102 (49%)	
Previous AF ablation	0		4 (3%)	
CKD	20 (27.8%)		112 (53.8%)	
Previous MI, peripheral artery disease, revascularization	17 (23.6%)		51 (24.5%)	
DM	22 (30.6%)		47 (22.6%)	
COPD	6 (8.3%)		26 (12.5%)	
Ischemic stroke/TIA	5 (6.9%)		17 (8.1%)	
Bleeding history with high risk for recurrence	3 (4.1%)		8 (3.8%)	
OSAS/CPAP users	3 (4.1%)/0		5 (2.4%)/1	
Significant liver dysfunction	0		3 (1.4%)	
Adherence to medication	25 (34.7%)		70 (33.7%)	
LVEF range				
HFpEF	22 (30.6%)		72 (34.6%)	
HFmrEF	13 (18.1%)		31 (14.9%)	
HFrEF	37 (51.4%)		99 (47.6%)	
Concomitant significant Right ventricular dysfunction	12 (16.7%)		59(28.4%)	
Isolated Right Heart Failure	1 (1.4%)		4 (1.9%)	
Signs on admission				
edema	33 (47.1%)		145 (72.5%)	
ascites	4 (5.7%)		24 (12.0%)	
Pleural effusion	25 (35.7%)		96 (48.0%)	
anasarca	7 (10.0%)		19 (9.5%)	
Pulmonary edema	29 (41.4%)		87 (43.5%)	
other	8 (11.4%)		2 (1.0%)	
Underlying triggering event				
Hypertensive emergency	28 (40.6%)		49 (24.1%)	
Acute coronary syndrome	15 (21.7%)		10 (4.8%)	
tachyarrhythmia	13 (18.8%)		36 (17.7%)	
Systemic infection	–		46 (22.7%)	
Acute kidney injury – cardiorenal syndrome	6 (8.7%)		56 (26.9%)	
New diagnosis of cardiomyopathy	12 (17.2%)		–	
New diagnosis of valvular disease (no endocarditis)	5 (7.2%)		–	
endocarditis	2 (2.9%)		–	
Non adherence to HF medication	–		55 (27.1%)	
Non adherence to diet/fluid restriction	–		67 (33.0%)	
other	9 (13%)		23 (11.3%)	
Laboratory investigations	Median (Q1, Q3)		Median (Q1, Q3)	
Left ventricular end diastolic diameter (mm)	54 (48, 60)		55 (48, 61)	
Left atrial diameter (mm)	45 (42, 48)		48 (44, 53)	
Pulmonary arterial systolic pressure (PASP) (mmHg)	45 (35, 57)		50 (41, 55)	
Urea (mg/dl)	51 (35, 86)		72 (50, 109)	
Creatinine (mg/dl)	1 (0.9, 1.5)		1 (1, 2.1)	
eGFR (ml/min/1.732)	48 (21, 71)		34 (17, 52)	
Potassium (mmol/L)	4 (3.9, 4.6)		4 (3.9, 4.8)	
Sodium (mmol/L)	140 (137, 142)		138 (134, 141)	
HbA1c (%)	6 (5.5, 6.2)		6 (5.8, 7.2)	
Hb (g/dL)	13 (11.4, 14.6)		12 (10.1, 13)	
BNP pg/ml	505 (347, 1372)		677 (335, 2000)	
proBNP pg/ml	6572 (1366, 14,765)		4337 (1828, 16,448)	
Ferritin μg/L	116 (40, 220)		97 (38, 189)	
CRP mg/L	12 (2, 19)		12 (3, 35)	
Inpatient treatment	Ν (%)		Ν (%)	
Intravenous vasodilators	6 (8%)		35 (16.8%)	
Vasopressors	5 (7%)		19 (9.1%)	
Intra-aortic balloon pump	0		1 (0.4%)	
Inotropes				
Levosimendan	11 (15.3%)	4 (5.5%)	46 (22.1%)	11 (5.2%)
Dobutamine		5 (7%)		24 (11.5%)
Dopamine		2 (2.8%)		11 (5.3%)
Renal replacement therapy				
Hemodialysis	4 (5.6%)	2 (2.8%)	14 (16.3%)	9 (4.3%)
Hemofiltration		1 (1.4%)		4 (1.9%)
Ultrafiltration		1 (1.4%)		1 (0.4%)
Oxygen supplementation				
Nasal canula	49 (68%)	40 (55.6%)	176 (84.6%)	137 (65.9%)
High flow nasal canula		3 (4.2%)		18 (8.7%)
Noninvasive ventilation		2 (2.8%)		15 (7.2%)
Mechanical ventilation		4 (5.5%)		6 (2.9%)
Electrical AF cardioversion	2 (2.8%)		2 (0.1%)	
Electrical VT cardioversion	2 (2.8%)		0	
Pharmaceutical AF cardioversion	2 (2.8%)		6 (2.9%)	
Intravenous iron replacement	4 (5.5%)		15 (7.2%)	
diuretics	64 (88.9%)		199 (95.7%)	
MRAs	31 (43%)		101 (48.6%)	
ACEi	20 (27.8%)		41 (19.7%)	
ARBs	21 (29%)		31 (14.9%)	
ARNI	7 (9.7%)		22 (10.6%)	
b-blockers	55 (76.4%)		156 (75%)	
SGLT2i	**14 (19.4%)**		**45 (21.6%)**	
CCBs—dihydropyridines	16 (22.2%)		36 (17.3%)	
CCBs—non dihydropyridines	3 (4.2%)		8 (3.8%)	
antiplatelets	30 (41.7%)		61 (29.3%)	
anticoagulants	19 (26.4%)		119 (57.2%)	
antidiabetics	16 (22.2%)		64 (30.8%)	
Digoxin	5 (6.9%)		5 (2.4%)	
Amiodarone	4 (5.5%)		18 (8.7%)	
Flecainide	0		1 (0.4%)	
Propafenone	1 (1.4%)		3 (1.4%)	
Ranolazine	0		1 (0.4%)	
Ivabradine	2 (2.8%)		2 (0.9%)	
Uric acid lowering drugs	7 (9.7%)		64 (30.8%)	
nitrates	5 (6.9%)		12 (5.8%)	
statins	31 (43%)		73 (35%)	
Proton pump inhibitors	44 (61%)		124 (60%)	
other	24 (33.3%)		84 (40%)	

**Table 6:** GDMT—chronic HF

	Number of prescribed GDMT categories					*P*
	0 (*N* = 24)	1 (*N* = 65)	2 (*N* = 80)	3 (*N* = 50)	4 (*N* = 16)	
Age	82 (72, 86)	82 (76, 87)	77 (68, 85)	75 (68, 85)	69 (64, 74)	< 0.001
Male Sex	33.3	41.5	33.3	23.4	21.4	0.29
BMI	26.1 (23.6, 31.1)	27.2 (24.2, 29,4)	26.1 (23.2, 30.9)	26.9 (25.6, 29.3)	27.1 (25, 31)	0.73
SBP	121 (110, 136)	127 (115, 138)	122 (110, 135)	120 (106, 132)	120 (110, 132)	0.40
HF type by EF						
HFrEF	70.8	73.8	75	70	81.3	0.91
HFmrEF	29.2	26.2	25	30	18.7	
HF cause						
Ischemic	50	33.8	46.3	46	43.8	0.012
DCM	4.2	3.1	3.8	18	25	
Valvular	29.2	30.8	20	20	18.8	
Hypertensive	16.7	15.4	20	8	0	
Other	0	16.9	10	8	12.5	
DM	36.4	38.1	45.5	54.2	68.8	0.13
CKD	47.8	44.6	53.8	52	37.5	0.69
AF history	29.2	56.9	55.7	53.1	50	0.19
Attended a HF unit	13	10.8	19.2	36	37.5	0.006
Prior HF hospitalization	33.3	47.6	59.5	58	60	0.16
MRA	–	15.4	35	92	100	< 0.001
ACEi/ARB	–	27.7	53.8	64	68.8	< 0.001
ARNI	–	0	13.8	26	31.3	< 0.001
Any RASi	–	27.7	66.3	90	100	< 0.001
BB	–	56.9	93.8	96	100	< 0.001
SGLT2i	–	0	5	22	100	< 0.001
LVEDD	57 ± 8	52 ± 8	56 ± 10	59 ± 9	61 ± 7	< 0.005

**Table 7:** GDMT per category—chronic HF

	BB		Any RASi		ARNI		MRA		SGLT2i		3 of GDMT		Reported adherence	
	OR	*p*	OR	*p*	OR	*p*	OR	*p*	OR	*p*	OR	*p*	OR	*p*
Age	0.972	0.127	0.984	0.262	**0.961**	**0.073**	**0.956**	**0.006**	**0.941**	**0.016**	.975	0.122	0.698	0.991
Male Sex	0.823	0.634	0.899	0.771	1.000	1.000	**0.512**	**0.098**	0.349	0.139	1.173	0.492	1.846	0.354
BMI	0.952	0.161	0.960	0.175	0.954	0.400	0.984	0.627	0.945	0.341	0.979	0.574	1.074	0.164
SBP	1.010	0.245	1.011	0.177	0.994	0.677	0.988	0.178	1.007	0.667	0.989	0.264	0.997	0.793
HFrEF (vs. HFmrEF)	1.106	0.808	1.125	0.742	0.546	0.255	0.839	0.653	**0.283**	**0.028**	0.958	0.923		
Attended a HF unit	0.965	0.945	1.880	0.138	1.787	0.314	**3.215**	**0.010**	**11.169**	**0.000**	**2.925**	**0.021**	0.465	0.533
Prior HF hospitalization	1.915	0.096	0.926	0.819	0.853	0.778	1.493	0.270	0.359	0.111	1.170	0.711	0.524	0.27
AF history	**2.098**	**0.056**	0.890	0.721	1.663	0.328	**3.434**	**0.001**	1.164	0.775	1.654	0.211	0.537	0.288
CKD	1.597	0.243	**0.392**	**0.008**	1.510	0.466	**0.523**	**0.097**	1.280	0.667	**0.385**	**0.034**	0.799	0.712
DM	0.965	0.926	1.405	0.304	1.523	0.414	**2.031**	**0.049**	**8.228**	**0.001**	1.45	0.349	0.586	0.348
HF etiology (vs. ischemic)		0.535		0.232		0.305		0.050		0.147				
DCM	2.388	0.293	1.418	0.569	2.182	0.245	**7.181**	**0.004**	**4.499**	**0.050**	**5.415**	**0.005**	**5.292**	**0.073**
Valvular	0.689	0.417	**0.443**	**0.048**	0.317	0.163	1.426	0.425	1.508	0.577	0.745	0.573	1.311	0.720
Hypertensive	1.015	0.981	0.765	0.617	0.695	0.688	1.611	0.414	0.499	0.590	0.743	0.685	3.327	0.175
Other	1.725	0.451	0.499	0.195	0.368	0.390	2.405	0.125	**5.837**	**0.065**	1.122	0.864	0.808	0.860

## Methods

The HECMOS study was organized by the 1 st Department of Cardiology of the National and Kapodistrian University of Athens (NKUA) in collaboration with the 2nd and 3rd NKUA Cardiology department under the auspices of INAKEN (Institute for study, research, education of vascular, heart, brain, and kidney nosologies). All Cardiology departments of the Greek National Public Health System as well as high volume ones of the private sector were invited to enrollment.

Data collection happened in a deanonymized manner and included patients’ demographics, physical characteristics and lifestyle parameters such as smoking habit, alcohol consumption and physical activity levels. Details regarding the participants’ past medical history were gained with emphasis on the presence of chronic HF, Atrial Fibrillation (AF), chronic kidney disease (CKD), Diabetes Mellitus (DM), hypertension, Covid-19 infection, stroke, severe liver dysfunction, bleeding history, chronic obstructive pulmonary disease (COPD), obstructive sleep apnea syndrome (OSAS) and intracardiac devices such as a pacemaker (PPM) or a defibrillator (IDC). Chronic HF cases were further interrogated about the left ventricular ejection fraction (LVEF) range, the right ventricular systolic capacity, the underlying substrate of cardiac dysfunction and any prior risk assessment for sudden cardiac death. Regular follow up in dedicated heart failure units was also noted if present. For all non-elective admissions, a detailed description was obtained which included symptoms and sings on admission as well as the working diagnosis on the day of the snapshot survey. Data collection included electrocardiographic, echocardiographic and laboratory results synchronous to the snapshot survey. Electrocardiographic features of interest included heart rate, heart rhythm, duration and morphology of QRS complex. Echocardiographic features of interest included LVEF, LVEDD, LA diameter, diameter of ascending aorta, RV function, estimation of the right ventricular systolic pressure and the presence of valvular prosthesis or of any valvular disease. Laboratory results included hemoglobulin, INR, uric acid, total cholesterol, HDL, LDL, triglycerides, iron studies, TSH, liver function studies, glycosylated hemoglobulin (HbA1 C), fasting glucose, maximum troponin, natriuretic peptides and high sensitivity CRP levels whereas serial values of urea, creatinine, sodium and potassium levels were also available. eGFR was calculated according to the 2021 CKD-EPI equation. A detailed medication and vaccination history was collected together with information about the participants’ adherence and any previous hospitalization events. Additionally, a comprehensive list of invasive or intravenous treatments administered during the index admission until the date of the survey was obtained together with all medications offered on the 3rd of March 2022.

## Definitions

In this study, HF definition and its further classification into the subtypes of HFrEF, HFmrEF, HFpEF and isolated right ventricular failure was in accordance to the latest ESC guideline (2021) [8]. Patients with acute HF with no previous history of HF were classified as new HF diagnosis cases. HF cases were further divided into groups with regards to the region of being hospitalized in. Centers located in Athens, Thessaloniki, Patras, Larisa, Heraklion, Ioannina and Alexandroupoli were classified as urban whereas the rest of the participating canters as rural. In the same lines, AF and acute coronary syndrome (ACS) cases were classified according to the 2020 respective ESC guidelines [9, 10]. Hypertension was defined in accordance to the 2018 ESH/ESC guidance [11]. The classification of DM and pre-DM was based on the WHO 2019 recommendations [12]. Finally, CKD definition in the current study required the presence of a stage 3 chronic kidney impairment (eGFR < 60 ml/min/1.73 m^2^) on at least two different measurements into a minimum period of 3 months prior to enrollment [13].

## Abbreviations

CVD: Cardiovascular disease
AF: Atrial fibrillation/PAF paroxysmal atrial fibrillation
ARNI: Angiotensin receptor neprilysin antagonist
b-blocker: Beta blocker
IHD: Ischemic heart disease
CKD: Chronic kidney disease
COPD: Chronic obstructive pulmonary disease
DM: Diabetes mellitus
eGFR: Estimated glomerular filtration rate
ESC: European Society of Cardiology
HF: Heart failure
HFmrEF: Heart failure with mid-range ejection fraction
HFpEF: Heart failure with preserved ejection fraction
HFrEF: Heart failure with reduced ejection fraction
BMI: Body mass index
ICD: Implantable cardioverter defibrillator
INAKEN: Institute for study, research, education of vascular, heart, brain, and kidney nosologies
GDMT: Guideline-directed medical treatment
SCD: Sudden cardiac death
DCM: Dilated cardiomyopathy
ACEi: Angiotensin converting enzyme inhibitor
ARB: Angiotensin receptor blocker
LVEDD: Left ventricular diameter in diastole
LVEF: Left ventricular ejection fraction
MRA: Mineralocorticoid receptor antagonist
NKUA: National and Kapodistrian University of Athens
OAC: Oral anticoagulant
OSAS: Obstructive sleep apnea syndrome
PA: Physical activity
PPM: Permanent pacemaker
RV: Right ventricle
SGLT2i: Sodium–glucose co-transporter 2 inhibitor
TIA: Transient ischemic attack
